# Fanconi syndrome and renal tubular necrosis in patients following ingestion of potentially contaminated red yeast rice supplement: Two case reports

**DOI:** 10.14814/phy2.70049

**Published:** 2024-09-10

**Authors:** Yoshiyuki Yoshikawa, Hitoshi Anzai, Kohei Odajima, Shinichiro Asakawa, Shigeyuki Arai, Osamu Yamazaki, Yoshifuru Tamura, Ryuji Ohashi, Shigeru Shibata, Yoshihide Fujigaki

**Affiliations:** ^1^ Department of Internal Medicine Teikyo University School of Medicine Itabashi‐ku Tokyo Japan; ^2^ Department of Integrated Diagnostic Pathology Nippon Medical School Bunkyo‐ku Tokyo Japan

**Keywords:** acute tubular necrosis, Fanconi syndrome, food supplement, foods with functional claims, red yeast rice

## Abstract

We present two cases of middle‐aged men who developed Fanconi syndrome and renal dysfunction after consuming “foods with functional claims (FFC)” containing red yeast rice. In the first case, the patient had consumed an FFC for 1 year and another FFC suspected to have contained nephrotoxin for 3 weeks; kidney biopsy performed during the acute phase of renal injury showed severe acute tubular necrosis and tubular cell regeneration. He achieved near‐complete recovery 40 days after the FFC was discontinued. In the second case, the patient had consumed FFC for 4 years and stopped 70 days prior to presentation; kidney biopsy revealed significant tubular recovery, persistent tubular injuries, and interstitial fibrosis. Although the manifestations of Fanconi syndrome subsided, mild renal dysfunction persisted. These cases suggest that FFC with nephrotoxins may induce Fanconi syndrome owing to acute tubular necrosis. Recovery is possible after discontinuing the FFC; while short‐term ingestion of FFC allows for tubular regeneration, its long‐term ingestion may cause irreversible damage and lead to chronic kidney disease. Long‐term follow‐up is crucial for preventing further renal deterioration.

## 1 INTRODUCTION

In late March 2024, several cases of illnesses, including renal damage, were reported due to the consumption of a health food product known as “foods with functional claims (FFC)” that contained beni‐koji (red yeast rice: RYR) produced by Kobayashi Pharmaceutical Co., Ltd. in Japan (Ministry of Health, Labor and Welfare). On March 22, the local government ordered the company to recall these products (Ministry of Health, Labor and Welfare). A report from the Japanese Society of Nephrology, based on a questionnaire administered to nephrologists, indicated that the individuals, likely affected by FFC with RYR, presented with Fanconi syndrome and acute tubulointerstitial injury since November 2023. Nephrotoxicity is suspected to have been caused by puberulic acid produced by contaminated blue mold and/or novel, unexpected compounds (Tanaka et al., 2024) in the FFC produced since at least June 2023 (Ministry of Health, Labor and Welfare). Although necrosis of the proximal tubules was found in animals treated with puberulic acid alone, further test results are awaited (Ministry of Health, Labor and Welfare).

In this report, we present two cases of Fanconi syndrome and renal dysfunction associated with the ingestion of the FFC with RYR. We discuss the possible clinicopathological course of the renal injuries by comparing the findings of these two cases with acute and resolution phases of the kidney injury.

## 2 CASE PRESENTATION

### 2.1 Case 1

A 43‐year‐old man had undergone a health check‐up 2 weeks before presentation, which showed normal urinalysis and a serum creatinine (sCr) of 0.9 mg/dL. He presented with epigastric discomfort and was prescribed rebamipide, a *Clostridium butyricum* combination drug, famotidine, and metoclopramide at a local clinic 6 days prior to admission. However, his symptoms persisted, and he revisited the clinic 1 day before presentation. His sCr was revealed to be 2.11 mg/dL, and he was admitted to our hospital. He had been consuming the FFC with RYR for 1 year and had began consuming the recalled products 3 weeks before presentation.

On admission, the patient was 173 cm tall, weighed 61 kg, had a blood pressure of 119/84 mmHg, and a body temperature of 36.9°C. Physical examination revealed no skin lesions or joint abnormalities. Laboratory examinations (Table 1) revealed heavy proteinuria, microscopic hematuria, elevated tubular injury markers, and renal dysfunction. We diagnosed the patient with Fanconi syndrome, which was indicated by probable renal tubular acidosis, hypophosphatemic hyperphosphaturia (TmP/GFR, 0.87 mg/dL; normal range 2.3–4.3 mg/dL), normoglycemic glucosuria, hypouricemic hyperuricosuria (FEUA 42.0%; normal range 5.5–11.1%), and aminoaciduria. The FFC was discontinued on the day of admission, and the patient's symptoms ameliorated.

**TABLE 1:** Laboratory data before and after ingesting the FFC (foods with functional claims) in Case 1 and Case 2.

		Case 1			Case 2		
		Day of hospitalization	40 days after stopping the FFC	82 days after stopping the FFC	First visiting our hospital	At biospy: 70 days after stopping the FFC	110 days after stopping the FFC
Urine	Reference						
Protein	(−)‐(±)	4+	(−)	(−)	2+	(−)	(−)
Glucose	(−)	3+	(−)	(−)	4+	(−)	(−)
Occult blood	(−)	1+	1+	1+	2+	(−)	(−)
Red blood cell	0‐4/F	5‐9/F	1‐4/F	1‐4/F	5‐9/F	0‐1/F	0‐1/F
Protein (g/gCr)	<0.3	9.54	0.05	0.06	1.31	0.03	0.02
Creatinine (mg/dL)		161.5	153.6	120.8	83.2	88.6	40.8
Uric acid (mg/dL)		58.8	83.9	76.5	41	42	18.5
Na (mEq/L)		116	112	125	145	104	43
K (mEq/L)		49	78	52	30	34	19
Cl (mEq/L)		156	145	138	177	89	42
Ca (mg/dL)		10.6	7.2	6.4	22.5	13.2	6.8
P (mg/dL)		76.6	89.8	80.7	49.1	35.5	11.3
NAG (U/L)	0.7–11.2	76.2	13.7	8.6	23.6	3.6	1.7
α1‐microglobulin (mg/L)	1.0–5.0	277.62	7.57	9.15	174.07	7.99	3.04
β2‐microglobulin (μg/L)	0–150	21,618	268	312	69,934	582	110
Amino aciduria		Positive				Positive	
Blood chemistry							
Albumin (g/dL)	4.1–5.1	3.7	4.6	4.5	4.2	3.9	4.3
Blood urea nitrogen (mg/dL)	8–20	15.9	17.3	21	11.2	16.8	14.4
Creatinine (mg/dL)	0.65–1.07	1.96	1	0.9	1.41	1.25	1.3
Uric acid (mg/dL)	3.7–7	1.7	4.2	5.2	1.6	4.6	5.5
Glucose (mg/dL)	8–20	89			106		
Na (mEq/L)	138–145	138	140	140	140	142	142
K (mEq/L)	3.6–4.8	3.4	4,2	4.2	3.7	4.1	4.4
Cl (mEq/L)	101–108	111	104	106	109	105	103
Ca (mg/dL)	8.8–10.1	8.8	9.5	9.1	9.4	9.5	9.8
P (mg/dL)	2.5–4.5	1.8	3.3	3.4	1.7	3.2	2.7
Mg (mg/dL)	1.8–2.4	2	2	2.2	2.5	2.1	2.2
Bicarbonate (mEq/L)	21–29	18.1	26.1	25.2	20.3	26.1	28.6
Anion Gap		8.9	9.9	8.8	10.7	10.9	10.4
eGFR (mL/min/1.73^2^)		31.6	65.9	74	42.4	48.4	46.3

**FIGURE 1:**
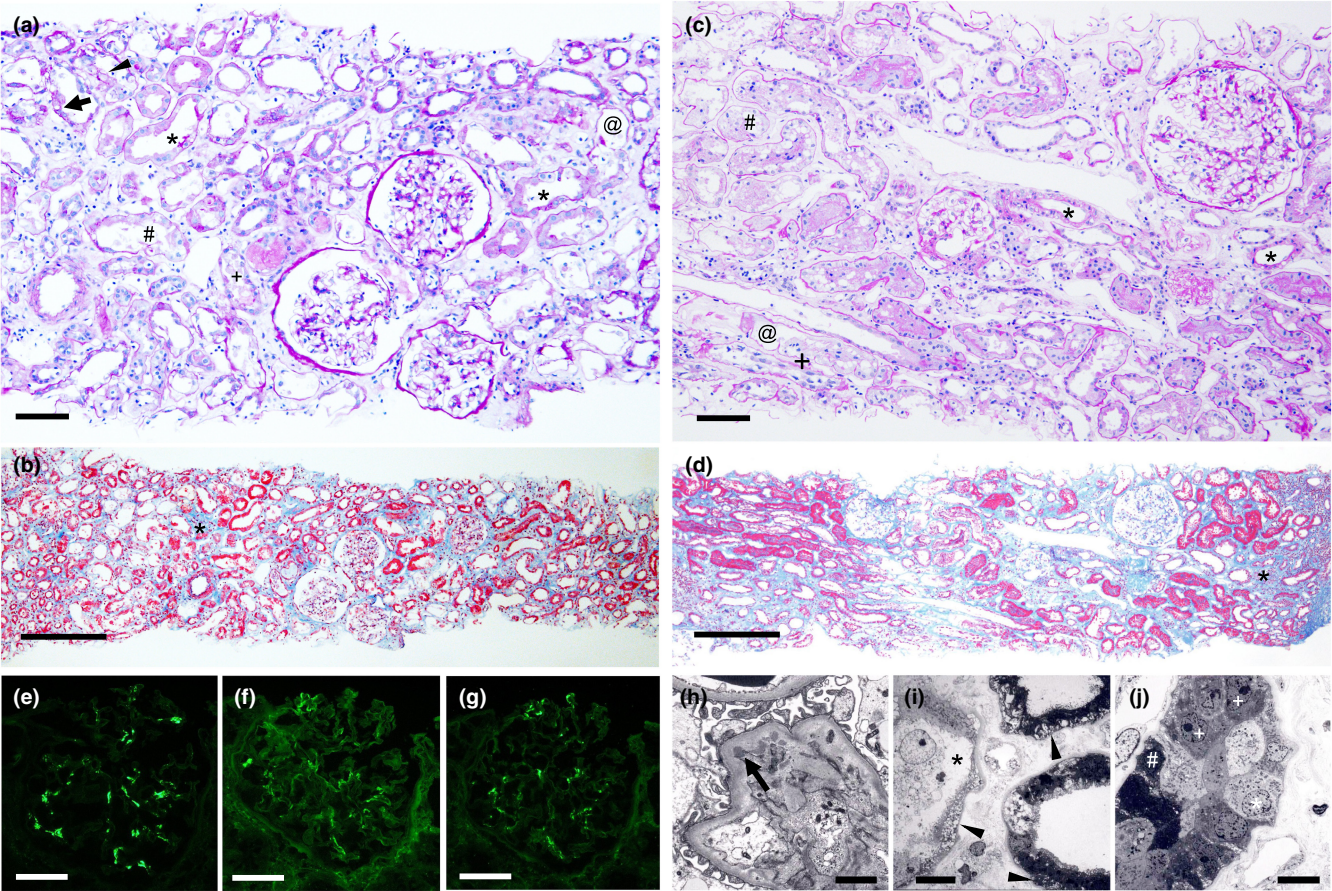
Kidney biopsy findings on light microscopy in Case 1 (a, b) and Case 2 (c, d). (a) Mild mesangial cell increase is seen in the glomeruli. Tubular dilatation with reduced brush borders (asterisks), cytoplasmic swelling (arrowhead) and vacuolation (arrow), tubular cell necrosis (hash mark), loss of epithelial cells (at mark), and cellular debris (plus) are found mainly in the proximal tubules. Periodic acid‐Schiff staining. Original magnification ×200. Bar = 100.0 μm. (b) Mild mononuclear cell infiltration (asterisk), and edema and slight fibrosis (blue color) are observed in the tubulointerstitium. Masson staining. Original magnification ×100. Bar = 200.0 μm. (c) Glomeruli show minor abnormalities. Some proximal tubules exhibit tubular injuries characterized by tubular dilatation with reduced brush borders (asterisks), tubular cell necrosis (hash mark), and loss of epithelial cells (at mark). Periodic acid‐Schiff staining. Original magnification ×200. Bar = 100.0 μm. (d) Some proximal tubules appear almost intact, mostly in clumps. Focal chronic inflammatory cell infiltration (asterisk) and focal interstitial fibrosis (blue color) are observed. Masson staining. Original magnification ×100. Bar = 200.0 μm. (e–g) Immunofluorescent findings in Case 1. Mildly positive for IgA (e), and weakly positive for κ (f) and λ light chains (g) in mesangial areas. (e–g); Bars = 50.0 μm. (h–j) Electron microscopy findings in Case 1. Small electron‐dense deposits (arrow) are observed in the mesangium without apparent foot process effacement (h). Intracytoplasmic electron‐lucent vesicles (arrowheads) and reduced brush borders are found in three proximal tubules, with one showing severe atrophy (asterisk). Edematous tubulointerstitial spaces are seen (i). Swollen (asterisk), atrophied (hash mark), and regenerating cells (pluses) are observed within the same proximal tubule (j). (h); Bar = 2.0 μm, (i), (j); Bars = 10.0 μm.

**FIGURE 2:**
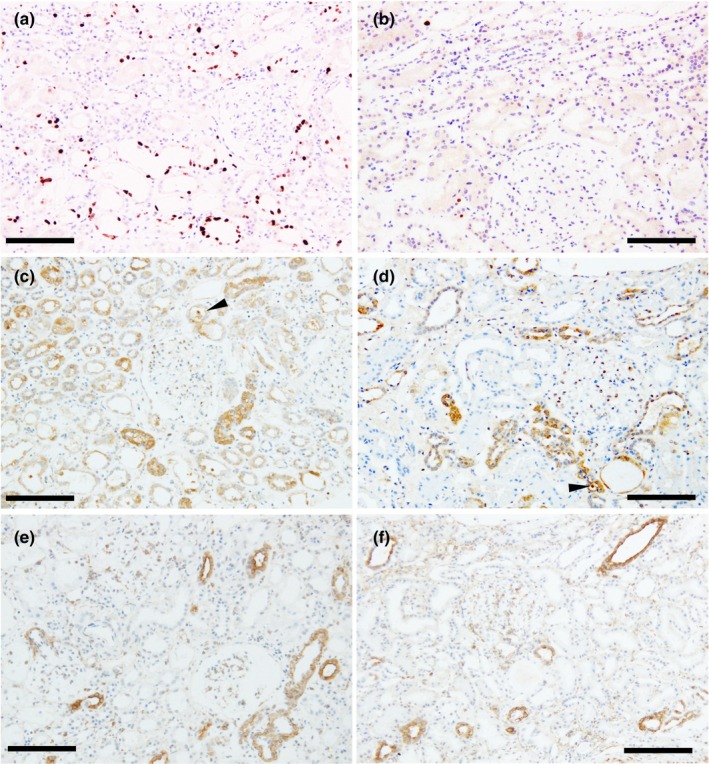
Immunohistochemistry findings in Case 1 (a, c, e) and Case 2 (b, d, f). (a) Many Ki67‐positive regenerating cells (brown color) are seen within the injured tubules in Case 1. (b) Ki67‐positive regenerating cells (brown color) in the damaged tubules are extremely scarce in Case 2. (c, d) Staining for the sodium chloride cotransporter (NCC) (brown color) is almost preserved in both Case 1 and Case 2, except for the occasional desquamated NCC‐positive cells (arrowheads). (e, f) Staining for aquaporin 2 (AQP2) (brown color) is almost preserved in both Case 1 and Case 2. A murine monoclonal anti‐human Ki67 antibody (clone MIG‐1, Dako Denmark A/S, Glostrup, Denmark), rabbit anti‐human NCC (Shibata S. et al. Proc Natl Acad Sci USA. 2014,111:15556–61), and goat anti‐human AQP2 antibody (Santa Cruz Biotechnology, Santa Cruz, CA) were used as primary antibodies. Sections were then incubated with peroxidase‐labeled anti‐mouse, anti‐rabbit, or anti‐goat antibody (Histofine Simplestain Max PO; Nichirei). Bars = 100.0 μm.

Kidney biopsy performed on the third day of admission revealed a mild increase in the number of mesangial cells and matrix expansion in some of the 45 obtained glomeruli (Figure 1a). Severe acute tubular injuries, mainly in the proximal tubules, were characterized by tubular dilatation with a low brush border, cytoplasmic swelling and vacuolation, tubular cell necrosis, epithelial cell loss, and cellular debris (Figure 1a). Mild mononuclear cell infiltration, 10%–15% edema, and slight fibrosis were observed in the tubulointerstitium (Figure 1a,b). The immunofluorescence study revealed 1+ IgA, trace κ light chain, and trace λ light chain in mesangial areas (Figure 1e–g). Staining for other immunoglobulins and complement was negative. Electron microscopy revealed small mesangial electron‐dense deposits (EDD) without apparent foot process effacement (Figure 1h). Some proximal tubular cells showed intracytoplasmic electron‐lucent vesicles, reduced brush borders, and severe atrophy (Figure 1i). Swollen, atrophied, and regenerating cells were also observed within the same proximal tubules (Figure 1j). Tubulointerstitial spaces were edematous (Figure 1i). Immunohistochemistry revealed numerous Ki67‐positive regenerating cells within the injured tubules (Figure 2a). Immunohistochemistry for sodium chloride cotransporter (NCC), a marker of the distal convoluted tubule (Figure 2c), and aquaporin 2 (AQP2), a marker of the collecting duct (Figure 2e), showed that both tubular segments were unaffected, except for occasional desquamated NCC‐positive cells. The patient was diagnosed with severe acute tubular necrosis (ATN) and tubular cell regeneration associated with reactive tubulointerstitial inflammatory cell infiltration and IgA nephropathy.

Forty days after discontinuing the FFC, his urinalysis normalized, and the Fanconi syndrome resolved (Table 1). His sCr decreased to 0.9 mg/dL 82 days after stopping the FFC (Table 1). IgA nephropathy was considered for follow‐up without medication.

### 2.2 Case 2

A 54‐year‐old man presented with 2+ proteinuria, 2+ occult blood in urine, 4+ glucosuria, sCr of 1.75 mg/dL, uric acid 1.5 mg/dL, blood glucose 102 mg/dL, and HbA1C 5.8% at a health check‐up approximately 3 months before presentation. He was referred to a local hospital, where he was suspected of having Fanconi syndrome.

The patient was then referred to our hospital and presented with proteinuria, hematuria, glucosuria, elevated tubular injury markers, and renal dysfunction, with a sCr of 1.41 mg/dL (Table 1). He was diagnosed with Fanconi syndrome, which was characterized by probable renal tubular acidosis, hypophosphatemic hyperphosphaturia (TmP/GFR, 0.87 mg/dL), normoglycemic glucosuria, and hypouricemic hyperuricosuria (FEUA, 43.4%) (Table 1). He had started taking FFC with RYR 4 years prior and had started recalling products a few months ago. The patient was instructed to discontinue the FFC.

The patient was admitted to our hospital 69 days after the initial visit. On admission, he was 172.4 cm tall, weighed 71 kg, had a blood pressure of 130/76 mmHg, and a body temperature of 36.7°C. The patient did not present with any specific findings. Most abnormal laboratory findings resolved upon admission, except for renal dysfunction and newly discovered amino aciduria (Table 1).

A kidney biopsy performed 70 days after discontinuing the FFC revealed one globally sclerosed glomerulus and other minor glomerular abnormalities among the 30 glomeruli obtained (Figure 1c). Over 50% of the proximal tubules appeared almost intact in clumps (Figure 1c,d). Other proximal tubules showed tubular injuries, characterized by tubular dilatation with a low brush border, tubular cell necrosis, and loss of tubular cells (Figure 1c). Focal chronic inflammatory cell infiltration and 5%–10% focal interstitial fibrosis were observed (Figure 1d). Atherosclerotic changes were observed in some of the arterioles and small arteries. Immunofluorescence staining was negative for all immunoglobulins and complements. Ki67‐positive cells in the damaged tubules were extremely scarce, indicating irreversible damage to tubular cells (Figure 2b). Immunohistochemical staining for NCC (Figure 2d) and AQP2 (Figure 2f) indicated that both tubular segments were preserved. The diagnosis was the resolution of ATN with incomplete tubular regeneration accompanied by focal interstitial fibrosis.

One hundred and ten days after stopping FFC, the Fanconi syndrome had almost resolved, but his sCr remained at 1.3 mg/dL.

## 3 DISCUSSION

Here, we present two cases of men with Fanconi syndrome and renal dysfunction caused by ATN. They did not consume any other potential nephrotoxins or concurrent medications known to cause ATN nor did they exhibit volume depletion. In both cases, the onset of kidney injury was temporally associated with the ingestion of an FFC that contained RYR, and clinical resolution occurred after discontinuation of the FFC, as reported by hazard reports from the Ministry of Health, Labor, and Welfare (Ministry of Health, Labor and Welfare).

In case 1, a kidney biopsy was performed during the acute phase of kidney injury, shortly after the patient stopped ingesting the supplement. In case 2, a biopsy was conducted during the resolution phase, 70 days after the patient stopped consuming the supplement. Fanconi syndrome and renal dysfunction improved after discontinuation of the FFC, consistent with reports from two studies (Miyazaki et al., 2024; Oda et al., 2024).

Fanconi syndrome impairs normal proximal tubule function, causing the urinary waste of substances reabsorbed at this site, including amino acids, low‐molecular‐weight proteins, phosphate, bicarbonate, glucose, and urate (Hall et al., 2014; Hall & Unwin, 2019). Although the proximal tubule also reabsorbs significant amounts of sodium, potassium, chloride, magnesium, and calcium, these losses can be compensated for by alternative uptake pathways in the distal tubule if they are intact (Hall et al., 2014). In our cases, Fanconi syndrome was observed; however, only a slightly low serum potassium level and hypophosphatemia were observed in case 1. This was likely due to the relatively preserved distal tubule function in both cases. Nearly intact immunostaining for NCC and AQP2 suggested that the distal parts of the tubules were not affected. However, because injured distal tubules do not express these biomarkers, these findings are inconclusive.

Our patients exhibited significant proteinuria, including tubular proteinuria, particularly in Case 1. This cannot be explained solely by the elevated levels of low‐molecular‐weight proteins observed in Fanconi syndrome. Electron microscopy revealed almost no foot process effacement, suggesting that humoral factors did not induce podocytopathy. Latent IgA nephropathy and/or the toxic effects of FFC on the glomerulus might have contributed to the transient massive proteinuria observed in case 1.

ATN can cause Fanconi syndrome, primarily due to insults targeting either endolysosomes or mitochondria (Hall & Unwin, 2019). In drug‐induced Fanconi syndrome, renal function recovery can sometimes take months and may not always be complete even after the therapy has stopped (Skinner, 2011). It remains unclear why some patients develop Fanconi syndrome because of drug toxicity, whereas others do not; thus, pharmacogenomics may play a role (Hall et al., 2014). In general, drug toxicity in the proximal tubule is dose‐related, which might also be true in cases involving the FFC.

ATN and active recovery during the acute phase of tubular injury were observed in Case 1. However, in Case 2, despite the recovery of renal function and resolution of Fanconi syndrome, while half of the tubules recovered, slight tubular necrosis and epithelial cell loss persisted, with almost no regenerating cells, even 70 days after stopping FFC. Additionally, focal interstitial fibrosis developed. The difference in the duration of FFC ingestion might explain the disparity in severity between cases. This suggests that more severe tubular injury occurred in case 2, leading to irreversible tubular injury and chronic interstitial fibrosis. However, it is unknown whether tubulointerstitial damage persisted in Case 1. It is necessary to explore whether there are differences in the nephrotoxic phenotype caused by FFC and the mode of renal recovery after FFC discontinuation. Understanding the molecular mechanisms underlying Fanconi syndrome is crucial.

To summarize, while Fanconi syndrome and renal function can improve after discontinuation of an FFC containing RYR, its pathology in case 2 suggests that tubular cell recovery may be incomplete, and chronic interstitial fibrosis may develop, leading to a transition to chronic kidney disease. Long‐term follow‐up is crucial to prevent further deterioration of renal function.

## AUTHOR CONTRIBUTIONS

Yoshiyuki Yoshikawa, Hitoshi Anzai, Kohei Odajima, Shinichiro Asakawa, Shigeyuki Arai, Osamu Yamazaki, Yoshifuru Tamura, and Ryuji Ohashi analyzed and interpreted the data. Shigeru Shibata and Yoshihide Fujigaki wrote the manuscript. All authors read and approved the final manuscript.

## FUNDING INFORMATION

No funding information provided.

## CONFLICT OF INTEREST STATEMENT

All the author declare that they have no conflicts of interest.

## ETHICS STATEMENT

Not applicable.

## PATIENT CONSENT STATEMENT

The patients have provided permission to publish these features of their cases, and the identity of the patients have been protected.

## Data Availability

The datasets used and/or analyzed during the present study are available from the corresponding authors upon reasonable request.
